# Bioconversion of Biomass-Derived Phenols Catalyzed by *Myceliophthora thermophila* Laccase

**DOI:** 10.3390/molecules21050550

**Published:** 2016-04-27

**Authors:** Anastasia Zerva, Nikolaos Manos, Stamatina Vouyiouka, Paul Christakopoulos, Evangelos Topakas

**Affiliations:** 1 Biotechnology Laboratory, School of Chemical Engineering, National Technical University of Athens, 5 Iroon Polytechniou Str., Zografou Campus, Athens 15780, Greece; natassaz@gmail.com (A.Z.); nickgmanos@gmail.com (N.M.); 2 Laboratory of Polymer Technology, School of Chemical Engineering, National Technical University of Athens, 5 Iroon Polytechniou Str., Zografou Campus, Athens 15780, Greece; mvuyiuka@central.ntua.gr; 3 Biochemical and Chemical Process Engineering, Division of Sustainable Process Engineering, Department of Civil, Environmental and Natural Resources Engineering, Luleå University of Technology, Luleå SE-97187, Sweden; paul.christakopoulos@ltu.se

**Keywords:** laccase, enzymatic oligomerization, catechol, pyrogallol, gallic acid

## Abstract

Biomass-derived phenols have recently arisen as an attractive alternative for building blocks to be used in synthetic applications, due to their widespread availability as an abundant renewable resource. In the present paper, commercial laccase from the thermophilic fungus *Myceliophthora thermophila* was used to bioconvert phenol monomers, namely catechol, pyrogallol and gallic acid in water. The resulting products from catechol and gallic acid were polymers that were partially characterized in respect to their optical and thermal properties, and their average molecular weight was estimated via solution viscosity measurements and GPC. FT-IR and ^1^H-NMR data suggest that phenol monomers are connected with ether or C–C bonds depending on the starting monomer, while the achieved molecular weight of polycatechol is found higher than the corresponding poly(gallic acid). On the other hand, under the same condition, pyrogallol was dimerized in a pure red crystalline compound and its structure was confirmed by ^1^H-NMR as purpurogallin. The herein studied green synthesis of enzymatically synthesized phenol polymers or biological active compounds could be exploited as an alternative synthetic route targeting a variety of applications.

## 1. Introduction

Recently, phenol polymers have attracted increasing attention as novel materials with various useful properties and applications. Research efforts are mainly focusing on the manufacture of novel polyphenols as an alternative to phenol-formaldehyde resins, widely used as adhesives, electric coatings and composites [1]. Despite their numerous applications, the present phenol-formaldehyde resin manufacturing processes pose many issues. Chemical synthesis of such products requires the use of inorganic catalysts, often of high cost and toxicity, and is usually carried out under extreme conditions of pressure, temperature and pH [2]. Thus an alternative procedure might be preferable, with low cost, environmentally friendly catalysts, carried out in milder conditions. Enzymes derived from various sources, from plants to microorganisms, can fulfil this need. Oxidoreductive enzymes, such as laccases [3], peroxidases [4,5], tyrosinases [6] or bilirubin oxidases [7] oxidize a variety of phenols, producing free radicals that are further subject to spontaneous polymerization. Laccases in particular are extensively studied in regard to phenols polymerization [2,3,8,9,10]. Laccase-synthesized phenol polymers have been tested in numerous applications, such as hair [11] or textile dyeing [3,10,12,13]. Some polyphenols seem to possess antioxidant properties that may be useful in the food, cosmetics or pharmaceutical industry [14]. Others have been tested as possible biosensors, due to the fact that some polyphenols, such as polycatechol or polyhydroquinone exhibit a certain extent of electrical conductivity [15,16,17,18]. Most polymerization efforts so far have been realized with commercial laccase from *Trametes versicolor* [11,19]. However, other commercial laccases, possibly with superior properties are also available, such as laccase from *Myceliophthora thermophila* expressed in *Aspergillus oryzae* [3,10,14]. The main advantage of Ascomycete laccases in biocatalysis is their higher thermostability and stability in a greater pH range, in contrast to Basidiomycete laccases, and thus they might be useful in a greater variety of applications. The difference in the use of a different biocatalyst may lie either in the optimization of the process parameters (enzyme stability, operation in preferable conditions) or even in the properties of the final product (bonds formed, polymerization degree and molecular weight, or overall material properties, such as thermoresistance or electrical conductivity). In this view, it might be useful to test the performance of various enzymes from different sources and with distinct properties in order to optimize the polymerization process and explore the properties’s variability of the resulting products. For example, oligomers of naturally occurring phenols might possess properties of great significance, applicable in medical or other industries, as is the case of purpurogallin, a pyrogallol dimer with beneficial human health effects [20,21,22].

In the present work, the polymerization or dimerization potential of a *M. thermophila* commercial laccase was explored, in an effort to obtain new biomaterials and antioxidants for various applications following a green and sustainable process. The effect of pH and temperature conditions for the synthesis of polycatechol and purpurogallin were investigated, aiming in optimizing the reaction conditions. The optimal conditions were retained for the bioconversion of gallic acid aiming in the biocatalytic production of novel polymers and antioxidants. The polymers produced were partially characterized in respect to their spectral and thermal properties. The dimerization of pyrogallol by the commercial laccase synthesized purpurogallin is a direct and mild process resulting in a pure compound without the need of any purification steps. The results of the study illustrate the superiority of enzyme biocatalysts in the production of materials and compounds from biomass derived phenols on behalf of the biorefinery concept.

## 2. Results and Discussion

### 2.1. Effect of pH and Temperature in Phenol Bioconversion

**Figure 1:**
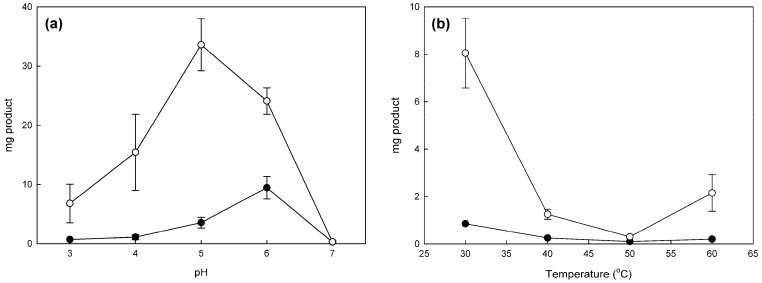
Effect of (**a**) pH and (**b**) temperature in polymerization reactions of catechol (**black circles**) and pyrogallol (**white circles**) from *M. thermophila* laccase under standard conditions performed in 25 mL reactions. Error bars represent the standard deviation from duplicate reactions.

In order to find the functional conditions where the enzyme catalyzes the oxidative bioconversion of various phenols, we studied the effect of pH and temperature for the polymerization of catechol and pyrogallol dimerization. The best conditions for *M. thermophila* laccase reactions were found similar in pH (pH 6 for catechol polymerization and pH 5 for pyrogallol dimerization), while in both reactions 30 °C was found as the optimal temperature, despite the thermophilic nature of the biocatalyst (Figure 1). This result might be due to the higher solubility of dioxygen, a necessary co-substrate for laccase catalysis, in lower temperatures. Similar conditions (pH 6, 30 °C) were applied for the bioconversion of gallic acid, as no study of the optimal conditions was carried out due to the low conversion yield obtained.

The dry powder resulting from the polymerization of catechol was insoluble in water, phosphate buffer pH 6 and acetic acid, but also in a number of organic solvents, such as ethanol, methanol, acetonitrile, acetone, *n*-hexane and methyl ethyl ketone. From the tested solvents, polycatechol was found to be soluble only in DMSO, tetrahydrofuran and dimethylformamide, and to a lesser extent, at 1 N NaOH. The subsequent bioconversion reactions were performed at pH 6 and 30 °C.

### 2.2. Preparative Synthesis and Product Characterization 

The 100 mL reaction mixtures yielded 105 mg, 142 mg, and 18.6 mg of dry powder for polycatechol, purpurogallin and poly(gallic acid) respectively, after 24 h at 30 °C. The corresponding yields for the two polymers were 26.3% and 4.7% for polycatechol and poly(gallic acid), respectively, while for the bioconversion of pyrogallol it was 35.5% with regard to the precipitate formed. Contrary to previous enzymatic phenol polymerization attempts using high redox potential Basidiomycete laccases, in the present study the use of organic solvents was not necessary, either during the reaction [2], or for the isolation of the final products [23].

**Figure 2:**
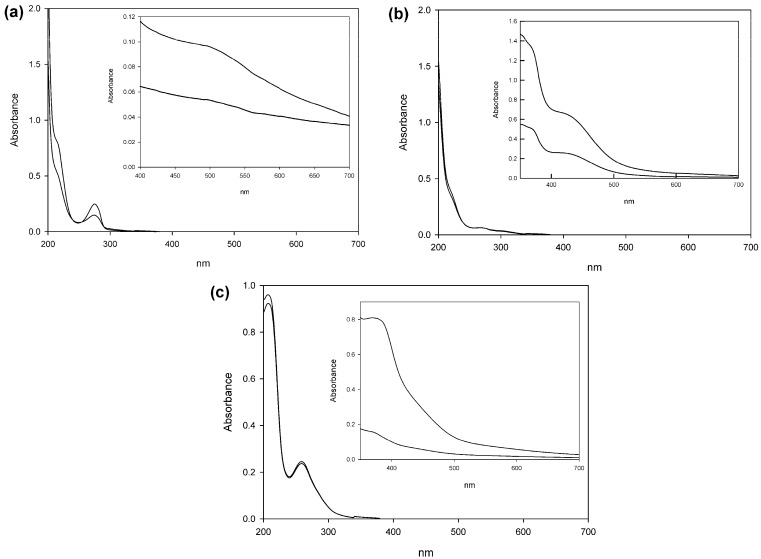
UV-Vis spectra of polymerization or dimerization products with catechol (**a**); pyrogallol (**b**); and gallic acid (**c**) as starting monomers. Grey lines represent the control reactions where laccase was substituted with heat-inactivated enzyme, while black lines represent the reaction mixtures after 24 h. The UV-Vis spectra presented correspond to 100-fold diluted samples, while in the inlets the spectra were recorded without any dilution.

All reaction mixtures changed colour from colourless to dark brown during oxidation of catechol and pyrogallol, and to black during oxidation of gallic acid, as is often reported for the enzymatic oxidation of phenolic compounds [3]. UV-Vis spectra were measured for all the reactions against their respective controls (Figure 2). Polycatechol absorbance is consistently higher than the control in the visible spectrum, presenting a wide peak around 463 nm. Polymerized gallic acid presents a significant increase in absorbance at 385 nm compared to the control. Purpurogallin presents peaks at the same wavelengths as the control in the ultraviolet area of the spectrum, but also shows two distinctive peaks in 370 and 430 nm.

The weight of the products measured corresponds only to the insoluble polymers or dimers that precipitate out of the reaction solution. This could partly explain the low yields obtained, especially in the case of gallic acid polymerization, seeing that the soluble poly- or oligomers are not measured in the precipitate, but rather are responsible for the colour of the reaction mixture, as indicated by the UV/Vis spectra. Possible differences in colour could be used as a means to quantify these soluble poly- or oligomers in the reaction supernatant. Colour darkening during the course of the reaction could also be attributed to the quinones formed by the action of the enzyme [24]. In the above described experiments it is apparent that the use of organic solvents was not required in the reaction mixture, contrary to previous studies [25,26].

### 2.3. Spectroscopic Analysis of Products

**Figure 3:**
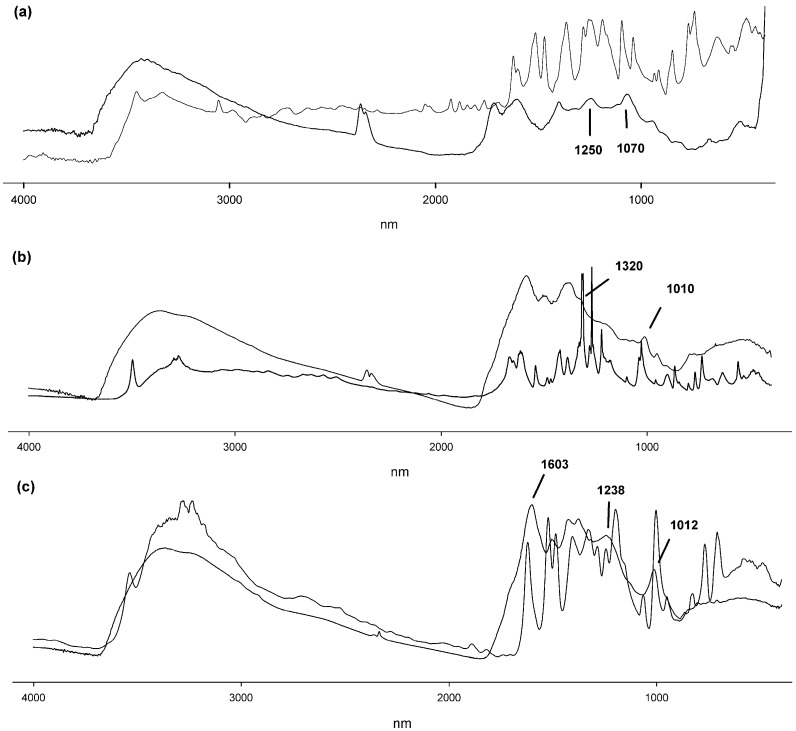
FT-IR spectra of the synthesized products and the respective monomers, (**a**) catechol; (**b**) gallic acid; (**c**) pyrogallol. Grey lines represent the monomer spectra, black lines represent the polymers. Characteristic peaks and shoulders, as referred to in the text, are indicated.

The FT-IR spectra of all three products were considerably smoother, probably due to the limited mobility of the side substituents especially in case of polymers, leading to a more rigid final product (Figure 3). In all three cases the sharp multiple monomer bands in the range 3000–3600 nm, usually attributed to the phenolic –OH moieties, were replaced by a broader band around 3400 nm in the polymer spectra. This is often observed in the case of phenol polymers and is attributed to the higher rigidity of the polymer, due to intramolecular hydrogen bonding, and to the limited mobility of the remaining free hydroxyl groups [25]. Polycatechol presents characteristic peaks in either ends of the 1000–1300 cm^−1^ range, consistent with the existence of C–O–C bond, as reported previously [3,25,26]. More specifically, for polycatechol, the peaks at 1250 and 1070 nm clearly indicate the presence of phenyl ether bonds, as previously described (Figure 3a; [26]).

**Figure 4:**
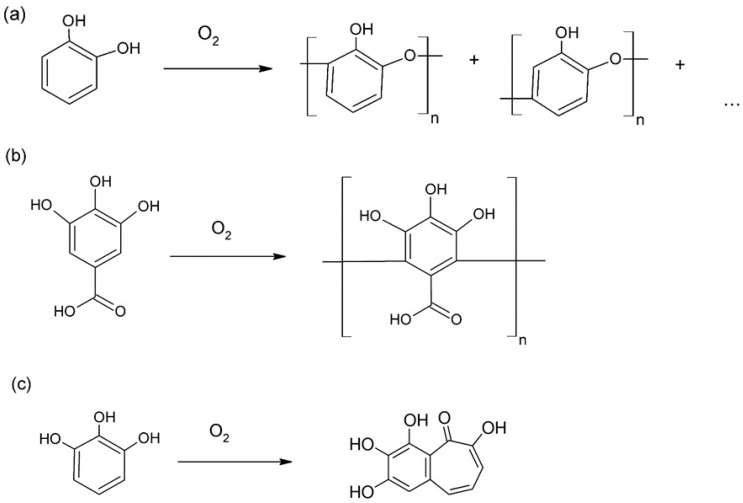
Enzymatic polymerization of (**a**) catechol; (**b**) gallic acid and dimerization of (**c**) pyrogallol by the commercial laccase from *M. thermophila*.

The band at 1606 nm could be attributed to the C=O bond of the quinone resulting from laccase oxidation. Characteristic peaks in the polymerized gallic acid spectrum correspond to wavelengths of 1586, 1500, 1378 nm, a shoulder at 1320 nm, and 1010 nm (Figure 3b). As previously described [26], peaks that could be attributed to the existence of C–O–C bonds were not observed, leading to the conclusion that the gallic acid monomers are joined with C–C bonds. The FT-IR spectrum of the pyrogallol bioconversion product is shown in Figure 3c. The C=O bond of the quinone formed from laccase oxidation of pyrogallol is possibly represented by the peak at 1603 nm. The bands observed correspond to those previously reported for purpurogallin, a pyrogallol dimer [20], namely 3381, 1374, 1238 and 1012 nm. The ^1^H-NMR spectra for the laccase catalysed synthesized polymers confirmed the above mentioned characterization, while in the case of pyrogallol dimerization to purpurogallin the purity and structure of the final product was identified. The proton NMR spectrum of the polycatechol product presents multiple peaks in the aromatic area between 6.2 and 7.5 ppm (Figure S1). Combining the ^1^H-NMR, FT-IR spectra together with literature data, we hypothesize a complex structure that could be a combination of proposed structures found in previous studies, as shown in Figure 4a [3,18].

**Figure 5:**
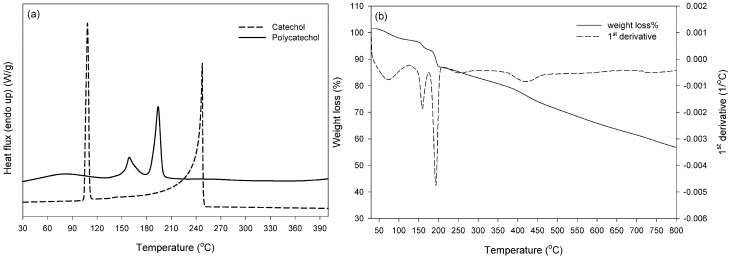
Thermal analysis curves (**a**) DSC and (**b**) TGA of enzymatically synthesized polycatechol.

In case of poly(gallic acid), the commercial laccase from *M. thermophila* was capable of catalyzing only the polymerization through C–C coupling of the aromatic chain, as previously reported [26]. This is clearly observed from the ^1^H-NMR spectrum where no signals were detected in the aromatic proton area, compared to the monomer of gallic acid where a singlet was observed at 6.81 ppm corresponding to two aromatic protons (data not shown). The structure elucidated in Figure 5b is identical to the poly(gallic acid) produced by oxidative polymerization of *T. versicolor* laccase, resulting in a semiconducting material [26]. On the other hand, pyrogallol was dimerized by *M. thermophila* laccase resulting in purpurogallin shown in Figure 5c. The dimerization product precipitated and the starting material was identified in the supernatant, therefore washing the crystal product with water yielded a pure biologically active compound. The pure compound was dissolved in DMSO-*d_6_* and analyzed by ^1^H-NMR. The corresponding spectrum differs considerably to the monomer of pyrogallol where only two peaks were detected at 6.32 ppm (t, *J* = 8.0 Hz, 1H) and 6.15 (d, *J* = 8.0 Hz, 2H) due to the symmetric structure of the aromatic compound (spectrum not shown). As illustrated in the ^1^H-NMR spectrum (Figure S2), the aromatic protons were replaced by alkenyls as revealed by their corresponding coupling constants at 7.25 ppm (d, *J* = 11.4 Hz, 1H), 6.98 ppm (d, *J* = 9.4 Hz, 1H) and 6.64 (dd, *J* = 11.3, 9.5 Hz, 1H). The single aromatic proton found in the structure of purpurogallin was detected at 6.80 ppm (s, 1H) as a broad singlet (Figure S2). The peaks observed in the spectrum of the pyrogallol polymerization product are consistent with those reported previously for pyrogallol dimer, purpurogallin [20]. Based on these results, in combination with data from related literature, we propose structures for these polyphenols or dimers, as presented in Figure 4.

### 2.4. Molecular Weight Distributions from GPC and Viscosity Measurements

In regard to molecular weight estimation, IV values measured in DMSO (30 °C) for polycatechol and poly(gallic acid) were 0.102 ± 0.013 dL·g^−1^ and 0.058 ± 0.002 dL·g^−1^, respectively. Polymerization rate appears to decrease as the repeating unit becomes more complex, with poly(gallic acid) presenting the lower yield and MW after 24 h of reaction. The viscosity results can be compared with the weight-average molecular weight ($\bar{M_{w}}$) values measured in GPC analysis. When comparing $\bar{M_{w}}$ values it can be seen, that the higher IV value (0.102 dL·g^−1^) of polycatechol corresponded to the higher $\bar{M_{w}}$ (26,240 Da) *vs.* the lower IV value (0.058 dL·g^−1^) of poly(gallic acid) and the lower $\bar{M_{w}}$ (7600 Da). Furthermore, polycatechol samples presented a polydispersity index (PDI) of 15.4, a significantly higher PDI value than previously reported. Most of the previous efforts concerning the enzymatic polymerization of catechol resulted in products of $\bar{M_{n}}$ up to 2660 Da with narrow polymer distribution, reflected by PDI values up to 1.2 [3,10,25], a discrepancy that can probably be attributed to the reaction conditions. Poly(gallic acid) samples, on the other hand, exhibited a $\bar{M_{n}}$ value of 2160 Da, with a PDI of 3.5, while it should be mentioned that this is the first report of a GPC analysis for enzymatically synthesized gallic acid polymers. Taking into account the molecular weight of the respective repeating units, the number-average degree of polymerization for the two polymers is calculated equal to 12.7 for poly(gallic acid) and 15.5 for polycatechol. The detailed results for all molecular weight determinations are presented in Table S1 in the Supplementary Materials.

### 2.5. Thermal Properties

The thermal properties of the synthesized products were investigated by differential scanning calorimetry and thermogravimetric analysis. Starting with the DSC results, analysis was also performed on the relevant monomers so as to monitor any residual amounts in the precipitated products. Catechol presented melting temperature at 106.2 ± 0.7 °C and boiling point at 244.6 ± 0.1 °C in agreement with literature values [27], while these peaks were not detected in the synthesized polymer. The formed polycatechol exhibited a broad endotherm in the range of 60–120 °C attributed to moisture/water evaporation, as well as two distinct endotherms: a small peak at 158.6 ± 0.3 °C and a main one at 193.4 ± 0 °C with total enthalpy (Δ*H*) at 239.1 ± 1.9 J·g^−1^ (Figure 5a).

**Figure 6:**
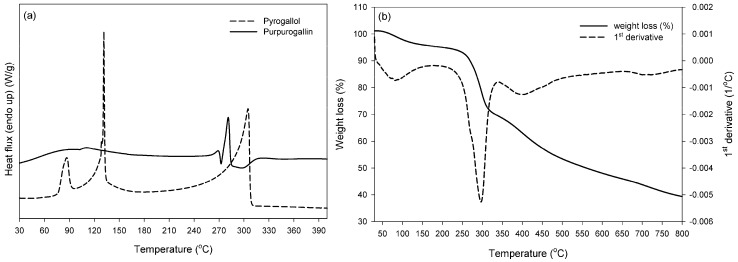
Thermal analysis curves (**a**) DSC and (**b**) TGA of enzymatically synthesized purpurogallin.

This double melting behavior can be a result of a bimodal distribution of crystal sizes, derived from different type or molecular-weight macromolecules, and/or from small or imperfect crystals [28,29,30]. DSC analysis of polycatechol has been also performed by Aktas *et al.* [25], who found a melting point at the lower temperature of 125 °C, probably due to the low number-average molecular weight (813 Da) of the relevant product. The DSC data were herein combined with TGA analysis, so as to evaluate polymer thermal resistance. Depending on the enzymatic polymerization conditions, differences in polycatechol TGA thermal behavior can be found in the literature [16,25,26]. In all cases there is a slight weight loss (<10%) up to 150 °C attributed to water, and then decomposition starts: the number of stages, the rate and the thermal stability vary with monomer and enzyme type. Accordingly, the herein synthesized polycatechol presented a slight weight loss (3%–4%) up to 140 °C, due to moisture and absorbed water (Figure 5b). Then, three stages of weight loss were observed, with maximum values at 157.6, 190.9 and 424.4 °C. The first two stages coincide with DSC endotherms, implying gravimetric effects (up to 13% weight loss) on melting: this can be attributed (i) to further loss of trapped solvent (water) immediately followed polymer melting; (ii) increasing vapour pressure of the volatile melt and/or (iii) partial decomposition of the polymer in the melt phase [31]. For temperatures above 200 °C, it is interesting to note that the degradation rate was relatively low compared to conventional polymeric materials, and the residue was found 57% up to 800 °C, slightly higher than the findings (65% mass loss) for laccase-synthesized polycatechol in the work of Aktas *et al.* [25]. It is important to note that in the aforementioned study the authors used a high redox potential laccase from *T. versicolor*. On the other hand, enzymatic synthesis with horseradish peroxidase [26], soybean peroxidase [26] and porphyrin [16] resulted in residues lower than 40%, implying the higher stability of the herein synthesized polycatechol. Similarily, in the case of purpurogallin (Figure 6a), the melting (131.7 ± 2.5 °C) and boiling (303.3 ± 0.4 °C) endotherms of the monomer (consistent with those reported by the supplier—Safety Data Sheet, product code 100612, Merck Millipore, Darmstadt, Germany) were not detected in the DSC curve of the synthesized product. Purpurogallin presented a melting endotherm at a higher temperature than polycatechol, *i.e.*, at *ca.* 280.15 ± 0.1 °C (Δ*H*_total_ = 103.2 ± 0.9 J·g^−1^). Regarding the TGA curve (Figure 6b), a slight weight loss was also found up to 140 °C due to moisture and absorbed water. Then, two stages of weight loss were seen, the first at 295.2 °C, *i.e.*, at higher temperature compared to polycatechol, and the second at 402 °C associated to degradation which resulted in total in residue less than 40% for temperature up to 800 °C. However, it should be noted that the weight loss upon purpurogallin melting (295 °C) was considerably higher (30%) than polycatechol (13%), indicating that gravimetric effects on melting can be mainly correlated to simultaneous dimer degradation obviously due to the higher melting temperature. Such thermal characteristic complicates the application of conventional melt polymerization techniques, and enhances the need for the development of alternative polymerization processes under mild conditions, as the enzymatic approach, which is applied in the current paper.

**Figure 7:**
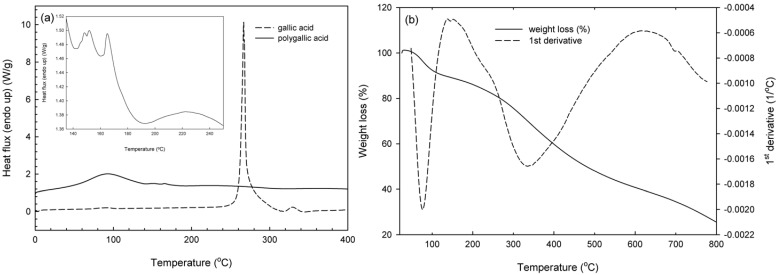
Thermal analysis curves (**a**) DSC and (**b**) TGA of enzymatically synthesized poly(gallic acid).

Finally, regarding poly(gallic acid) thermal behavior, monomer at 266 °C was not detected in the polymer samples (Figure 7a), while a broad endotherm was again shown in the area of 100 °C due to water/moisture evaporation. Small melting peaks appeared at 147 and 165 °C with no significant gravimetric effects in the TGA (Figure 7b). No sharp melting endotherm was evidenced as in the case of polycatechol, obviously due to its amorphous character in agreement also with the lowest molecular weight that it possessed. The degradation rate presented a maxiumum value at 331 °C resulting in 25% up to 800 °C. Again, however, it should be noted that slow degradation was observed, a result that confirms the relatively high thermal resistance of the enzymatically synthesized polymers. Especially in the case of gallic acid polymer, the remaining residue above 200 °C is over 80%, equally satisfactory with previously reported polymerization efforts with *T. versicolor* laccase [26].

## 3. Materials and Methods

### 3.1 Enzymes and Chemicals

All chemicals were purchased from either Applichem (Darmstadt, Germany) or Sigma-Aldrich (Taufkirchen, Germany) and were of the highest purity available. Pyrogallol was purchased from Merck Millipore (Darmstadt, Germany). For the polymerization reactions, a commercial laccase (multicopper oxidase EC 1.10.3.2) from *M. thermophila*, expressed in *Aspergillus oryzae*, named Novozym 51003, was kindly provided by Novo Nordisk A/S (Bagsvaerdt, Denmark). The commercial enzyme preparation was provided in liquid form, and according to the manufacturer, its activity was 1000 LAMU g^−1^.

### 3.2 Determination of Laccase Activity and Protein Concentration

Laccase activity was routinely estimated using 2,2′-azino-bis(3-ethylbenzothiazoline-6-sulphonic acid (ABTS) as the substrate. The reaction mixture contained 3 mM ABTS, a properly diluted enzyme sample and 100 mM phosphate buffer pH 6. After a 10 min incubation at 30 °C, the absorbance was measured at 420 nm, and the concentration of oxidized ABTS was calculated using ε_420nm_ = 36000 M^−1^·cm^−1^. Control reactions were also performed with thermally inactivated enzyme. One unit of laccase activity was defined as the amount of enzyme required to oxidize 1 μmol of ABTS per minute in the above conditions. The activity of the laccase preparation was found to be 134.58 ± 8.8 U·mL^−1^. Protein concentration was estimated using the Lowry method [32], using bovine serum albumin as the standard. The commercial preparation was found to contain 51.57 ± 2.31 mg·mL^−1^ protein.

### 3.3 Bioconversion Reactions

A typical polymerization/dimerization reaction was carried out as follows: in 250 mL Erlenmeyer flasks 89 Units mL^−^^1^ of commercial laccase and 400 mg substrate were added in 100 mL of sodium phosphate buffer 50 mM, pH 6. The reaction mixture was incubated for 24 h in a temperature-controlled shaking incubator, at 30 °C, 180 rpm, unless stated otherwise. The studied substrates were catechol, pyrogallol and gallic acid. Control reactions were carried out with heat inactivated enzyme, to eliminate any possible background oxidation of substrates. After 24 h, the mixture was filtered with a 0.2 μm hydrophilic polyethersulfone filter and the solids retained were washed with ultrapure water to remove enzyme and unreacted monomers. The reaction product was freeze-dried prior to analysis. For the optimization of the polymerization reaction conditions on different values of pH and temperature, reactions were carried out in smaller volumes (25 mL) using catechol or pyrogallol as the substrate. The optimum pH of polymerization reactions was investigated using the following buffers: 100 mM sodium phosphate–citrate buffer (pH 3–5), 100 mM sodium phosphate buffer (pH 6–7).

### 3.4 Product Analysis

UV-Vis spectra were recorded with a U-2900 UV-Visible spectrophotometer (Hitachi, Tokyo, Japan) in the range of 200–700 nm. FT-IR spectra were recorded with a Nicolet Magna-IR 560 Spectrophotometer (Thermo Fisher Scientific Inc., Waltham, MA, USA) in the 4000–400 cm^−^^1^ range in the form of KBr pellets. ^1^H-NMR analyses were recorded on an Avance III 600 spectrometer (Bruker, Billerica, MA, USA) with DMSO-*d*_6_ as solvent and TMS as an internal standard. DSC analysis was performed using a DSC 1 STARe System (Mettler, Greifensee, Switzerland) under nitrogen flow (10 mL·min^−1^). Approximately 5–6 mg of sample were subjected to heating from 0 °C to 400 °C at a rate of 10 °C·min^−1^ in duplicate runs. TGA was also conducted on a Mettler TGA/DSC1 thermobalance with a heating rate at 10 °C·min^−^^1^ from 30 to 800 °C under nitrogen flow (20 mL·min^−^^1^). Degradation temperatures (*T*_d_) were determined at the maximum weight loss. The intrinsic viscosity (IV, dL·g^−^^1^) was measured in DMSO at concentration of 0.2 g·dL^−^^1^ in an Ubbelohde capillary viscometer at 30 ± 0.1 °C. All samples were dissolved at room temperature and filtered prior measurement using filter paper. Measurements were performed in duplicates and IV values were determined using the Solomon-Ciuta equation (Equation (1)) [33]:

(1) $$\text{IV=}\sqrt{2\left(\frac{t}{t_{0}}-\text{ln}\frac{t}{t_{0}}-1\right)}$$

where, t is the flow time of solution and t_0_ is the flow time of pure solvent.

Gel Permeation Chromatography was carried out using a Shimadzu Analytical HPLC (Kyoto, Japan) equipped with a RID-10A-SPD M20A refractive index detector, and a PL Gel packed column (Agilent, Santa Clara, CA, USA). Samples were dissolved in tetrahydrofuran, and the injection amount of the sample was 5 μg. The flow rate was maintained at 0.75 mL·min^−1^ with a column temperature of 40 °C. A calibration curve of polystyrene with molecular weight ranged from 162 to 5,000,000 Da was used for the calculations of the average molecular weight and polydispersity.

## 4. Conclusions

The use of oxidative enzymes, such as laccases and peroxidases for synthetic applications is emerging as an attractive alternative to conventional chemical synthesis methods. On the other hand, such biocatalytic tools permit the biosynthesis of novel materials with relatively low financial and environmental costs. Especially for oxidative enzymes, their major advantage could be that they offer a means to exploit simple phenols as building blocks, a resource abundantly available through lignin-rich byproducts of various processes, such as the paper and pulp industry. In the present paper, we examined the potential of a thermostable commercial laccase for the polymerization and dimerization of biomass derived phenols and their characterization. Our results further support the prominence of enzymes as tools for polymer synthesis, due to the observation that the structure and physical properties of the desired material can be, at least to a certain extent, controlled by the choice of the monomer unit and/or the biocatalyst. Further work is needed in order to explore whether the final product and its physicochemical properties depends also on other factors, such as the choice of the solvent, the enzyme/substrate ratio, the duration of the reaction etc. Research in this direction could possibly lead to the production of custom-made materials and bioactive compounds with distinct properties for specific applications.

## Supplementary Materials

The following are available online at: http://www.mdpi.com/1420-3049/21/5/550/s1, Figure S1: ^1^H-NMR spectrum of polycatechol, Figure S2: ^1^H-NMR spectrum of purpurogallin, Table S1: GPC results and solution viscosity measurements for laccase-synthesized polycatechol and poly(gallic acid).
