# Supplementary material for: Bioconversion of Biomass-Derived Phenols Catalyzed by Myceliophthora thermophila Laccase
Source: Molecules. 2016 Apr 27;21(5):550. doi: 10.3390/molecules21050550 (PMC6273956; doi:10.3390/molecules21050550)
Supplement: Supplementary file 1 [file molecules-21-00550-s001.pdf]

Supplementary Materials: Bioconversion of Biomass Derived Phenols Catalyzed by *Myceliophthora thermophila* Laccase

Anastasia Zerva, Nikolaos Manos, Stamatina Vouyiouka, Paul Christakopoulos, Evangelos Topakas

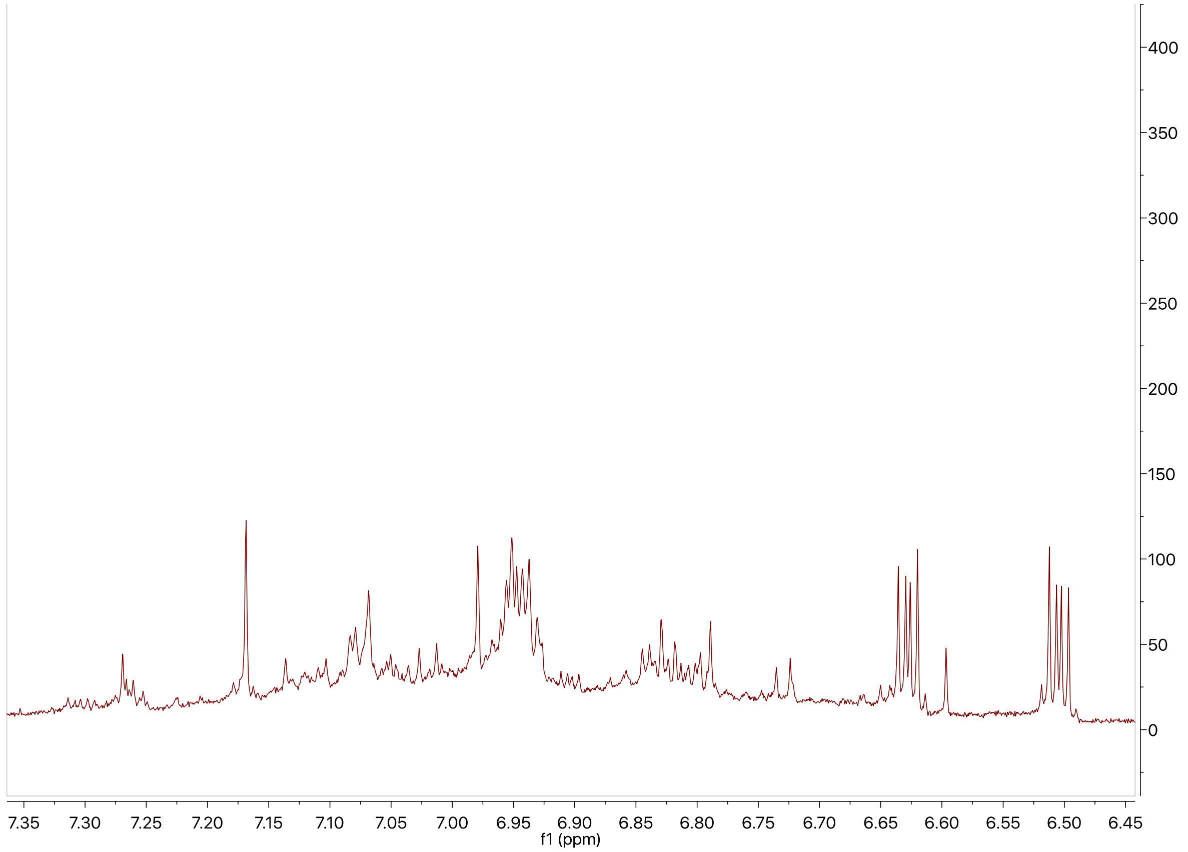

Figure S1. ^{1}H-NMR spectrum of polycatechol.

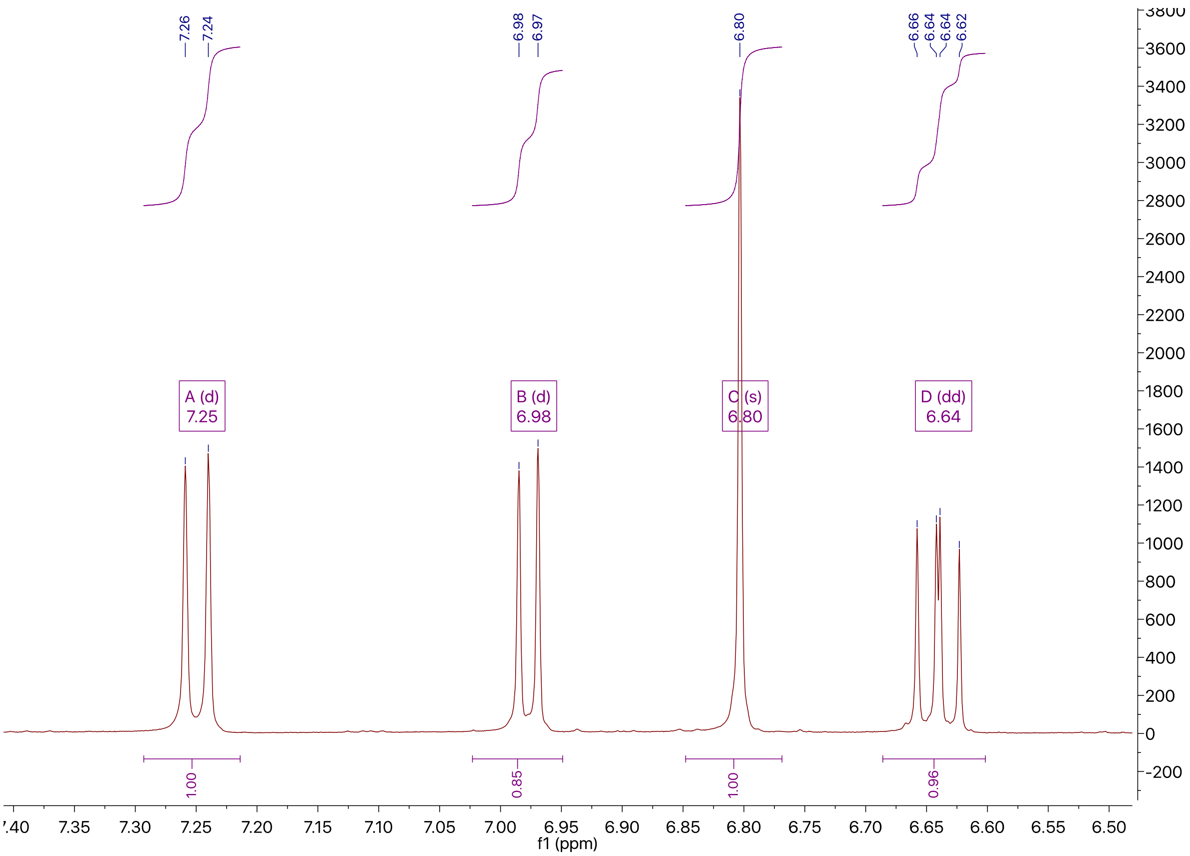

Figure S2. ^{1}H-NMR spectrum of purpurogallin.

Table S1. Results of GPC and solution viscosity measurements for laccase-synthesized polycatechol and poly(gallic acid).

	$\overline{M_n}$ *(Da)*	$\overline{M_w}$ *(Da)*	*PDI*	*IV (dL g*$^{-1}$*)*
Polycatechol	1700	26240	15.4	0.102 ± 0.013
Poly(gallic acid)	2160	7600	3.5	0.058 ± 0.002
